# MicroRNAs in hereditary and sporadic premature aging syndromes and other laminopathies

**DOI:** 10.1111/acel.12766

**Published:** 2018-04-25

**Authors:** Diane Frankel, Valérie Delecourt, Karim Harhouri, Annachiara De Sandre‐Giovannoli, Nicolas Lévy, Elise Kaspi, Patrice Roll

**Affiliations:** ^1^ Aix Marseille Univ INSERM MMG Marseille France; ^2^ APHM, Hôpital la Timone Service de Biologie Cellulaire Marseille France; ^3^ APHM, Hôpital la Timone Département de Génétique Médicale Marseille France

**Keywords:** aging, genetics, Hutchinson–Gilford progeria syndrome, laminopathies, lamins, microRNA

## Abstract

Hereditary and sporadic laminopathies are caused by mutations in genes encoding lamins, their partners, or the metalloprotease ZMPSTE24/FACE1. Depending on the clinical phenotype, they are classified as tissue‐specific or systemic diseases. The latter mostly manifest with several accelerated aging features, as in Hutchinson–Gilford progeria syndrome (HGPS) and other progeroid syndromes. MicroRNAs are small noncoding RNAs described as powerful regulators of gene expression, mainly by degrading target mRNAs or by inhibiting their translation. In recent years, the role of these small RNAs has become an object of study in laminopathies using *in vitro* or *in vivo* murine models as well as cells/tissues of patients. To date, few miRNAs have been reported to exert protective effects in laminopathies, including miR‐9, which prevents progerin accumulation in HGPS neurons. The recent literature has described the potential implication of several other miRNAs in the pathophysiology of laminopathies, mostly by exerting deleterious effects. This review provides an overview of the current knowledge of the functional relevance and molecular insights of miRNAs in laminopathies. Furthermore, we discuss how these discoveries could help to better understand these diseases at the molecular level and could pave the way toward identifying new potential therapeutic targets and strategies based on miRNA modulation.

**Abbreviations**
3′UTR: 3′ untranslated transcript region
ADLD: adult‐onset autosomal‐dominant leukodystrophy
AGO: Argonaute protein
APLD: acquired partial lipodystrophy
ASCs: adipose stem cells
AWS: atypical Werner syndrome
ceRNA: competing endogenous RNA
CMD: congenital muscular dystrophy
CMT2B1: Charcot–Marie–Tooth disease type 2B1
DCM1A: dilated cardiomyopathy type 1A
EDMD: Emery–Dreifuss muscular dystrophy
FPLD2: Dunnigan‐type familial partial lipodystrophy
HCV: hepatitis C virus
HGPS: Hutchinson–Gilford progeria syndrome
HGPS‐like: Hutchinson–Gilford progeria‐like syndromes
hMSCs: human mesenchymal stem cells
hsa: *Homo sapiens*
IGF‐1: insulin‐like growth factor‐1
INM: inner nuclear membrane
iPSCs: induced pluripotent stem cells
LAD: lamin‐associated domains
LGMD1B: limb‐girdle muscular dystrophy type 1B
LIRLLC: lipoatrophy with diabetes, hepatic steatosis, hypertrophic cardiomyopathy, and leukomelanodermic papules
lncRNA: long‐noncoding RNA
MAD: mandibuloacral dysplasia
MEFs: mouse embryonic fibroblasts
miRNAs: microRNAs
mmu: mus musculus
mRNAs: messenger RNAs
MSCs: mesenchymal stem cells
NETs: nuclear envelope transmembrane partner proteins
NLS: nuclear localization signal
NPC: nuclear pore complex
nt: nucleotides
ORF: open reading frame
RD: restrictive dermopathy
ROS: reactive oxygen species
VSMCs: vascular smooth muscle cells

## 1 LAMINS AND LAMINOPATHIES

Lamins are type V intermediate filament proteins specifically expressed in the nucleus of eukaryotic cells. They are divided into A‐type and B‐type families. *LMNA* encodes A‐type lamins produced by alternative splicing of its pre‐mRNA. The two major isoforms of this A‐type include lamin A and lamin C, but minor isoforms have also been described: lamin AΔ10, lamin C2 (Furukawa, Inagaki & Hotta, 1994; Lin & Worman, 1993; Machiels et al., 1996), and more recently LMNAΔ447 and LMNAΔ297 (DeBoy et al., 2017). Lamins A and C are mainly expressed in the nucleus of differentiated somatic cells. Concerning B‐type lamins, *LMNB1* encodes lamin B1, whereas *LMNB2* encodes lamins B2 and B3 by alternative splicing (Elkhatib et al., 2015; Furukawa et al., 1994; Feng Lin & Worman, 1995). Lamins B1 and B2 are expressed in nearly all somatic cells, while lamin B3 is a specific spermatid type.

B‐type lamins and the lamin A are produced as precursors forms called “prelamins,” which undergo several maturation steps before their importation into the nucleus. Firstly, the cysteine of the CAAX box in the C‐terminal domain of these prelamins is farnesylated by a farnesyltransferase. This process leads to the prelamin anchoring into the endoplasmic reticulum membrane on its cytosolic leaflet. Secondly, the AAX sequence is cleaved by FACE1/ZMPSTE24 or by FACE2/Rce1. Thirdly, the cysteine residue on which the farnesyl group was previously fixed is carboxymethylated by a isoprenylcysteine carboxyl methyltransferase (Cau et al., 2014). After this step, B‐type lamins are mature and remain farnesylated. As they carry a nuclear localization signal (NLS) in their tail domain, they are imported through the nuclear pore complex (NPC) and remain anchored in the inner nuclear membrane (INM) of the nuclear envelope. For this reason, mature lamins B (B1, B2 and B3) are only present in the nuclear *lamina*, a protein meshwork at the nuclear periphery, close to the INM. Prelamin A undergoes a fourth and last maturation step, corresponding to the cleavage of the 15 C‐terminal amino acids by the metalloproteinase FACE1/ZMPSTE24. Lamin C, a splicing variant of lamin A missing the 98 carboxy terminal amino acids, is directly translated into a mature protein. As mature lamin A and lamin C are not farnesylated, they are not directly anchored in a membrane. They bear an NLS leading to their importation into the nucleoplasm. In the nucleus, lamins A and C are constituents of the nuclear matrix: (i) the *lamina* where they interact with B‐type lamins, with several nuclear envelope transmembrane partner proteins (NETs) of the INM and with the NPC, and (ii) the internal nuclear meshwork forming a component of the nucleoskeleton (Cau et al., 2014; Gruenbaum & Foisner, 2015; Turgay et al., 2017). This lamin meshwork plays a major role in cell structure by conferring the architecture of the nucleoplasm and maintaining the nuclear shape (Ungricht & Kutay, 2017). Lamins are essential for chromatin organization via the lamin‐associated domains (LAD), which are genomic regions that make contact with the nuclear *lamina* (van Steensel & Belmont, 2017). Lamins are also involved in many nuclear functions, such as gene expression, DNA replication, and repair (Gruenbaum & Foisner, 2015; Naetar, Ferraioli & Foisner, 2017). A‐type lamins are involved in mechanosignaling and mechanosensing and contribute to nuclear stiffness, whereas B‐type lamins are involved in nucleus elasticity.

The importance of lamins in many physiological mechanisms explains the wild spectrum of diseases linked to these proteins. Hereditary laminopathies are therefore caused by mutations in genes encoding lamins (primary forms) or proteins implicated in their maturation, such as ZMPSTE24, and in genes encoding their partners (secondary forms; Schreiber & Kennedy, 2013; Worman, 2012; Worman & Bonne, 2007). Laminopathies can also be acquired. For example, they can have an iatrogenic origin, as in HIV patients treated with protease inhibitors. Most molecules of this class inhibit ZMPSTE24 function, resulting in the blockage of prelamin A maturation and its accumulation in nuclei, leading to clinical manifestations of lipodystrophy syndrome (Béréziat et al., 2011; Caron et al., 2007; Coffinier et al., 2008; Miranda et al., 2007).

The first hereditary laminopathy described was Emery–Dreifuss muscular dystrophy in 1999 (Bonne et al., 1999). Since then, the number of these diseases has increased. Mutations in *LMNA* or *ZMPSTE24* cause many different phenotypes, which can be classified in multisystem diseases or tissue‐specific phenotypes (Worman, 2012; Worman & Bonne, 2007). Table 1 summarizes this classification: The multisystem diseases correspond mostly to accelerated aging disorders including progeria and other progeroid syndromes, whereas the tissue‐specific diseases contain lipodystrophic syndromes, striated muscle diseases, and an axonal peripheral neuropathy. It is of note that overlapping phenotypes exist, such as mandibuloacral dysplasia, which is a combination of progeroid disorder and partial lipodystrophy (Agarwal, Kazachkova, Ten & Garg, 2008). Mutations in B‐type lamins have also been described, enlarging the field of laminopathies. Thus, heterozygous mutations in *LMNB2* have been reported to predispose to the development of acquired partial lipodystrophy (APLD), also called “Barraquer‐Simons syndrome” (Hegele et al., 2006), and duplication of *LMNB1* leads to adult‐onset autosomal‐dominant leukodystrophy (ADLD; Padiath et al., 2006).

**Table 1:** Classification of primary and ZMPSTE24‐related laminopathies

Diseases	Genes	Recurrent mutations	Transmission modes	Prenylated prelamin A	Clinical features	References
**Progeria and Progeroid syndromes**						
Hutchinson–Gilford progeria syndrome, typical form (HGPS)	*LMNA*	c.1824C>T; p.G608G	*De novo*; HET	Progerin (Prelamin AΔ50)	Early childhood: alopecia, narrow nasal bridge, receding mandible, loss of subcutaneous fat, progressive joint contractures, nail dystrophy, tightness skin, delayed tooth, eruption, osteoarthritis, arteriosclerosis leading to myocardial infarction or stroke. Overlapping phenotypes with MAD, RD	De Sandre‐Giovannoli et al. (2003) Eriksson et al. (2003)
Hutchinson–Gilford progeria‐like syndromes (HGPS‐like)	*LMNA*	c.1968G>A; p.Q656Q c.1968+1G>A c.1968+2T>C, c.1821G>A c.1968+5G> A, c.1868+1C>G	*De novo*; HET	Depending on mutation: Progerin ± Dermopathin (Prelamin AΔ90); Prelamin AΔ35	Depending on mutation: premature aged appearance in adulthood (c.1968G>A); Classical HGPS phenotype, neonatal forms (c.1968+1G>A and c.1821G>A); Short stature, progeroid appearance (c.1968 + 5G>A and c.1868+1C > G)	Barthélémy et al. (2015) Moulson et al. (2007) Harhouri et al. (2016)
Atypical progeroid syndromes	*LMNA*	p.D136Y, p.E138K, p.D300N, p.T528M, p.T528M + p.M540T	HET, HOM or compound HET	–	Depending on mutation: premature aged appearance with cases of classical HGPS phenotype; musculoskeletal features; early arteriosclerosis and cardiovascular events	Verstraeten et al. (2006) Doubaj et al. (2012) *Unpublished data*
Restrictive dermopathy (RD)	*LMNA ZMPSTE24*		*De novo*; HET *(LMNA)* HOM *(ZMPSTE24)*	Dermopathin (Prelamin AΔ90) Full‐length prelamin A	Intrauterine growth retardation, reduced fetal movements; thin, tightly adherent translucent skin, superficial vessels, facial dysmorphism, generalized joint ankylosis; death in the first week of life. Overlapping phenotypes described with HGPS	Navarro et al. (2004) Navarro et al. (2005)
Atypical Werner syndrome	*LMNA*		HET	–	Initial symptoms earlier than typical WS: cataract, dermatological pathology (scleroderma‐like skin…), short stature, graying or thinning of hair, diabetes mellitus type 2, hypogonadism, osteoporosis, osteosclerosis of digits, atherosclerosis, voice change	Chen et al. (2003) Renard et al. (2009)
Mandibuloacral dysplasia (MAD)	*LMNA* (MAD‐A) *ZMPSTE24* (MAD‐B)	p.R527H c.1085_1086insT + p.N265S	HOM *(LMNA)* Compound HET *(ZMPSTE24)*	Full‐length prelamin A in MAD‐B	Growth retardation, craniofacial anomalies, mandibular hypoplasia, osteolysis (clavicle and distal phalanges), pigmentary skin changes, lipodystrophy (normal or increased fat tissue in neck and trunk, loss in tissue from extremities) insulin resistant diabetes mellitus. Overlapping phenotypes described with HGPS	Novelli et al. (2002) Agarwal, Fryns, Auchus and Garg (2003) Ben Yaou et al. (2011)
**Lipodystrophy/atrophy syndromes**						
Dunnigan‐type familial partial lipodystrophy (FPLD2)	*LMNA*	Hotspot in 482 position: p.R482W/Q/L	HET	Full‐length prelamin A	Abnormal subcutaneous adipose tissue distribution: loss of fat from the upper and lower limbs, the gluteal and truncal localization, muscular appearance, accumulation of fat in face and neck (double chin). Insulin‐resistance, diabetes mellitus, *acanthosis nigricans*, hypertriglyceridemia.	Shackleton et al. (2000) Araújo‐Vilar et al. (2009)
Acquired partial lipodystrophy (APLD) or Barraquer–Simons syndrome	*LMNB2 (predisposing mutations)*		HET	–	Lipodystrophy with several subcutaneous fat loss affected regions (neck, arms, chest, face, abdomen), type IV or V dyslipoproteinemia, hypertension, hepatomegaly, hirsutism	Hegele et al. (2006)
Generalized lipoatrophy, insulin‐resistant diabetes, disseminated leukomelanodermic papules, liver steatosis, and cardiomyopathy (LIRLLC)	*LMNA*	p.R133L	HET	–	Hepatic steatosis, hypertriglyceridemia, insulin‐resistant diabetes, generalized atrophy of the subcutaneous fat, sunken cheeks, and muscular pseudohypertrophy of the four limbs, thin and atrophic skin on the back of the hands and feet, hyperelasticity, or joint mobility	Caux et al. (2003)
**Striated muscle diseases**						
Emery–Dreifuss muscular dystrophy type 2 and 3 (EDMD)	*LMNA*	At least 106 mutations published, the most frequents are p.R249Q, p.R453W	HET or HOM	–	Joint contractures starting in early childhood, slowly progressive muscle weakness (start in humeroperoneal then scapular and pelvic girdle muscles), cardiac manifestations (syncope, arrhythmias, dilated cardiomyopathy, congestive heart failure, etc.). Overlapping phenotypes with FPLD, CMT	Bonne et al. (1999) Raffaele Di Barletta et al. (2000)
Limb‐girdle muscular dystrophy type 1B (LGMD1B)	*LMNA*	At least 51 mutations published, mostly missense	HET	–	Limb‐girdle distribution of muscular weakness (starting in the proximal lower limb muscles then upper limb muscles), cardiac manifestations (atrioventricular cardiac conduction disturbances, dilated cardiomyopathy), the absence of early contractures	Muchir et al. (2000)
Dilated cardiomyopathy type 1A (CDM1A)	*LMNA*	At least 126 mutations published, mostly missense	HET	–	Systolic dysfunction, early conduction defects, arrhythmias, left ventricular dilatation, congestive heart failure, sudden cardiac death. Overlapping phenotypes described with FPLD	Fatkin et al. (1999)
Congenital muscular dystrophy (CMD)	*LMNA*	At least 23 published mutations, the most frequents are p.R249W, p.E358K	HET	–	Early infancy: dropped head syndrome, muscle axial and cervicoaxial weakness, severe hypotonia, delayed motor development, respiratory insufficiency	Quijano‐Roy et al. (2008)
Heart‐hand syndrome, Slovenian type	*LMNA*	c.IVS9‐12T>G (c.1609‐12T>G)	HET	–	Progressive cardiac conduction defect, tachyarrhythmia, dilated cardiomyopathy, brachydactyly (hand less affected than feet), muscle weakness	Renou et al. (2008)
**Peripheral neuropathy**						
Charcot–Marie–Tooth disease type 2B1 (CMT2B1)	*LMNA*	p.R298C	HET	–	Axonal peripheral neuropathy, distal muscle weakness and atrophy, depressed tendon reflexes, mild sensory loss	De Sandre‐Giovannoli et al. (2002)
**Other diseases**						
Adult‐onset leukodystrophy (ADLD)	*LMNB1*	Gene duplication	HET	–	Chronic progressive neurologic disorders: cerebellar, pyramidal, and autonomic abnormalities, symmetrical decreases in white‐matter density	Padiath et al. (2006)

Recurrent mutations, transmission modes, prelamin A production, and typical clinical features are described for each laminopathy together with the main references. HET, heterozygous; HOM, homozygous.

## 2 microRNAs

MicroRNAs (miRNAs) are small noncoding RNAs of 18–25 nucleotides (nt) in length. The first described was lin‐4 in *C. elegans* in 1993 (Lee, Feinbaum & Ambros, 1993). To date, the specific database miRbase lists 2,588 mature miRNAs for *Homo sapiens* (hsa; Griffiths‐Jones, Grocock, van Dongen, Bateman & Enright, 2006), and 66,160 articles are referenced in PubMed using the keyword “microRNA,” with a spectacular increase for 10 years. miRNA genes are present in intergenic regions with an independent promoter (52%), in introns (40%), or less frequently in exons (8%). In these last two cases, the expression of the miRNA is linked to transcription of the host gene (Hsu et al., 2006; Rodriguez, Griffiths‐Jones, Ashurst & Bradley, 2004). To become mature, miRNAs need to undergo several post‐transcriptional modifications. Initially, the miRNA is transcribed by RNA polymerase II as a large primary transcript (>1 kb) called “pri‐miRNA.” The pri‐miRNA is recognized by the microprocessor complex, which contains DGCR8 and the RNase III enzyme Drosha (Denli, Tops, Plasterk, Ketting & Hannon, 2004; Han et al., 2004). In the nucleus, Drosha cleaves the pri‐miRNA into a 70 nt stem‐loop precursor called “pre‐miRNA” (Lee et al., 2003). The pre‐miRNA is then exported to the cytoplasm using exportin 5 and the small Ran GTPase (Bohnsack, Czaplinski & Gorlich, 2004; Yi, Qin, Macara & Cullen, 2003). In the cytoplasm, the pre‐miRNA is therefore cleaved by the endonuclease Dicer into a ≈ 25 nt duplex miRNA (Hutvágner et al., 2001). This duplex is bound by the Argonaute protein (AGO) in a complex called “RNA‐induced silencing complex” (RISC), which eliminates one strand of the duplex to conserve the guide strand corresponding to the mature ≈25 nt miRNA (Kobayashi & Tomari, 2016). This mature miRNA, retained in RISC, recognizes the target transcripts by complementarity to the seed region of the miRNA. This 5′ region of the miRNA, corresponding to 2 to 7 nt, is essential to target messenger RNAs (mRNAs), mostly in their 3′ untranslated transcript region (3′UTR). More rarely, this targeting could also be localized in the 5′UTR or in the open reading frame (ORF) of mRNAs (Bartel, 2009; Helwak, Kudla, Dudnakova & Tollervey, 2013). The interaction between the miRNA and the target mRNA forms a Watson–Crick pair, resulting in the inhibition of protein synthesis, depending on the pairing of the miRNA and its target. If the pairing is perfect, AGO generates an endonucleolytic cleavage leading to mRNA degradation. If mismatches are present, the mRNA can be deadenylated and then degraded or undergoes translational repression (Jonas & Izaurralde, 2015; Pasquinelli, 2012). Even if miRNAs are mostly described to repress gene expression, miRNAs can also upregulate expression of target genes (Mortensen, Serra, Steitz & Vasudevan, 2011; Oldenburg et al., 2017; Vasudevan, Tong & Steitz, 2007). Moreover, miRNAs can regulate gene expression at the transcriptional level after their nuclear reimportation and hybridization in promoter regions of these genes (Place, Li, Pookot, Noonan & Dahiya, 2008). For these reasons, miRNAs are currently known as powerful regulators of gene expression. One miRNA has the ability to interact with many target mRNAs and to regulate their expression (Gennarino et al., 2012; Helwak et al., 2013; Hendrickson et al., 2009; Tsang, Ebert & van Oudenaarden, 2010). Currently, 64% of the human miRNAs belong to seed families, which correspond to microRNAs sharing the same or a highly similar seed region (Kozomara & Griffiths‐Jones, 2011). As the seed region is essential to recognize mRNA, several microRNAs can target the same mRNA and therefore act together to modulate the expression of this mRNA (Guo, Ingolia, Weissman & Bartel, 2010; Hausser & Zavolan, 2014; Hendrickson et al., 2009). Since their discovery, miRNAs have been described in an impressing list of physiological and pathological pathways (Hammond, 2015). For example, miRNAs are central players during development (e.g., neural or cardiac; Abernathy & Yoo, 2015; Porrello, 2013; Reinhart et al., 2000) or immunity (Zhu, Pan & Qian, 2013). They play an important role in all cellular processes, such as cell proliferation (Johnson et al., 2007; Navarro & Lieberman, 2010), differentiation (Navarro & Lieberman, 2010), and apoptosis (Ghodgaonkar et al., 2009). They have been largely studied in cancer, in which they are described to function as oncogenes or tumor suppressor genes and are grouped under the term “oncomiRs” (Bracken, Scott & Goodall, 2016; Hayes, Peruzzi & Lawler, 2014). Several miRNAs are considered organ/tissue‐specific; for example, miR‐122 is liver‐specific (Lagos‐Quintana et al., 2002), and miR‐1, miR‐206, and miR‐133 families are called “myomiRs” (Mok, Lozano‐Velasco & Münsterberg, 2017; van Rooij, Liu & Olson, 2008). Moreover, in some cases, miRNAs have been associated with genetic diseases: A mutation of the miR‐24 target site in *Slitrk1* leads to Tourette syndrome, whereas a mutation in the seed region of miR‐96 leads to autosomal‐dominant nonsyndromic hearing loss (Bandiera, Hatem, Lyonnet & Henrion‐Caude, 2010). As in all these fields, miRNAs have also been studied in laminopathies. In the next sections, we will present the current knowledge of the different miRNAs linked to laminopathies (Table 2).

**Table 2:** Summary of miRNAs studied in *in vitro* or *in vivo* models of laminopathies and other lamin‐related models

Laminopathies	microRNAs	Targets	Models	References
**Progeria and progeroid syndromes**	miR‐9	*LMNA*	HeLa cells MEF (*Lmna* ^HG/+^, wild‐type) *Lmna* ^plao‐5nt^ knock‐in mice *Lmna* ^plao‐utr^ knock‐in mice Human iPSC and derived cells	Jung et al. (2012) Jung et al. (2014) Nissan et al. (2012)
	miR‐29	*Ppm1d*	Mouse fibroblasts (*Zmpste24* ^*−*/*−*^, wild‐type)	Ugalde, Español et al. (2011) and Ugalde, Ramsay et al. (2011)
	miR‐1	*IGF1*	Liver tissues from WT and progeroid mice Cultured fibroblasts derived from patients with HGPS	Mariño et al. (2010)
	miR‐365	*Rasd1*	MEF (*Zmpste24* ^*−*/*−*^, wild‐type)	Xiong et al. (2015)
	miR‐342‐5p	*GAS2*	MEF (*Zmpste24* ^*−*/*−*^, wild‐type)	Zhang et al. (2017)
	miR‐141‐3p	*ZMPSTE24*	hMSC Wild‐type mice	Yu et al. (2013)
**Lipodystrophy**/**atrophy syndromes**	miR‐335	*FXR1*	FPLD2 fibroblasts Primary adipose stem cells (*LMNA* p.R482W)	Oldenburg et al. (2017)
	miR‐141‐3p	*ZMPSTE24*	Fibroblasts (*LMNA* p.R482W, p.D47Y, p.R133L) VSMC (*LMNA* p.R482W, p.D47Y, p.R133L)	Afonso et al. (2016)
	miR‐140	NEAT1 (lncRNA)	Primary adipocyte‐derived stem cells (miR‐140 knock‐out mice)	Gernapudi et al. (2016)
**Striated muscle diseases**	miR‐100, miR‐192, miR‐335	*PPP3CA*,* NFAT5* and *Sp1*	Muscular biopsies of patients (*LMNA* p.R60G, p.R294Q, p.R321X, p.R377C)	Sylvius et al. (2011)
	miR‐1, miR‐130a, miR‐133a, miR‐133b, miR‐146b, miR‐151‐3p, miR‐200a, miR‐339‐3p		*Lmna* ^H222P^ knock‐in mice	Vignier et al. (2013)
**Peripheral neuropathies**	/	/	/	/
***LMNB1***‐**related diseases**	miR‐23a	*LMNB1, PTEN* 2700046G09Rik (lncRNA)	Wild‐type mice Mice overexpressing mmu‐miR‐23 Senescent fibroblasts	Lin et al. (2013) Lin and Fu (2009) Lin et al. (2014) Dreesen et al. (2013)
	miR‐31	*Cdkn2a*	*Lmnb1* ^Δ/Δ^ MEFs	Malhas et al. (2010)

hMSCs, human mesenchymal stem cells; MEFs, mouse embryonic fibroblasts; iPSCs, induced pluripotent stem cells; HGPS, Hutchinson–Gilford progeria syndrome; FPLD2, Dunnigan‐type familial partial lipodystrophy, type 2; VSMCs, vascular smooth muscle cells; lncRNA, long‐noncoding RNA.

## 3 miRNAs IN HEREDITARY LAMINOPATHIES

### 3.1 miRNAs in accelerated aging disorders (Figure 1)

**Figure 1:**
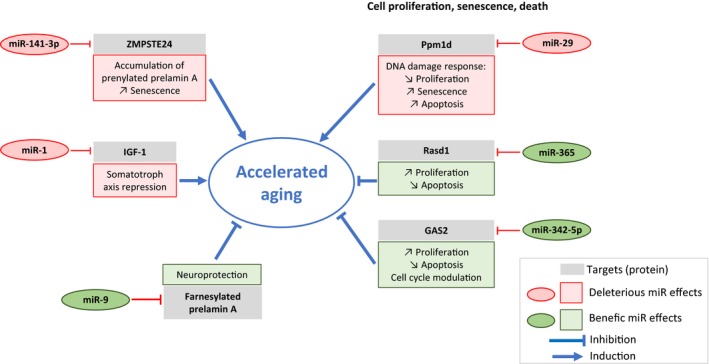
MicroRNAs potentially implicated in the pathophysiology of laminopathies associated with accelerated aging. This figure is proposed according to the results obtained on *in vitro* and *in vivo* models of accelerated aging syndromes (Jung et al., 2012, 2014; Mariño et al., 2010; Nissan et al., 2012; Ugalde, Español et al., 2011; Ugalde, Ramsay et al., 2011; Xiong et al., 2015; Yu et al., 2013; Zhang et al., 2017)

In its classical form, Hutchinson–Gilford progeria syndrome (OMIM: #176670) is caused in the vast majority of cases by the *LMNA* c.1824C>T (p.G608G) mutation localized in exon 11. This mutation leads to the activation of a cryptic splicing site in exon 11, leading to the loss of 150 nucleotides with the conservation of the reading frame. The resulting truncated protein, called “progerin,” lacks 50 amino acids compared to lamin A localized on its carboxyl‐terminal domain and including the cleavage site of the FACE1/ZMPSTE24 endoprotease (De Sandre‐Giovannoli et al., 2003; Eriksson et al., 2003). Consequently, progerin remains farnesylated on the cysteine at the C‐terminal end and anchored to membrane bilayers. The protein is still imported into the nucleus as the NLS is not removed or inactivated by the mutation and finally stays anchored to the INM. Progerin accumulation in the *lamina* leads to nuclear shape deformations that are associated with nuclear dysfunctions. Clinically, patients suffer from accelerated aging symptoms, such as skeletal alterations, muscular dystrophy and atrophy, cutaneous changes, and cardiovascular complications, which generally lead to myocardial infarction or stroke (Ullrich & Gordon, 2015). The average lifespan of these patients is 14.6 years. Interestingly, patients with progeria who present a multisystemic disease do not have cognitive deterioration. Fong and colleagues studied the expression of lamins in the brains of various mouse models. They used mice producing only lamin A (*Lmna* ^LAO/LAO^ knock‐in mice), prelamin A (*Zmpste24* ^−/−^ mice that produce prenylated prelamin A but no mature lamin A), and nonfarnesylated progerin (*Lmna* ^nHG/nHG^ knock‐in mice, which yield exclusively nonfarnesylated progerin from mutant prelamin A transcripts). They demonstrated that lamin A or prelamin A/progerin was dramatically reduced in the brain compared to lamin C (except of course for *Lmna* ^LAO/LAO^ knock‐in mouse model; Jung et al., 2012). They showed that expression of lamin A or its derivatives (prelamin A and progerin) was restricted to vascular and meningeal cells. Indeed, even in *Lmna* ^LAO/LAO^ mice, the level of lamin A was extremely low in the brain compared to other organs. They identified a brain‐specific miRNA, miR‐9, as the major regulator of lamin A expression in this organ. Using different *in vitro* models (HeLa cells, wild‐type, and *Lmna* ^HG/+^ mouse embryonic fibroblasts transfected with miR‐9; Jung et al., 2012), and *in vivo* models, *Lmna* ^plao‐5nt^ knock‐in mice (in which a 5 nt mutation was introduced into the predicted miR‐9 binding site of prelamin A 3′UTR) and *Lmna* ^plao‐utr^ mice (in which prelamin A 3′UTR was replaced by lamin C 3′UTR; Jung et al., 2014), they demonstrated that miR‐9 regulates lamin A or prelamin A expression in the central nervous system by directly targeting their common 3′UTR on mRNAs. These results were confirmed by Nissan et al. (2012) using *in vitro* models of neural precursors and neurons of patients with HGPS. They developed an induced pluripotent stem cell (iPSC) model generated from dermal fibroblasts, which they secondarily derived in neural stem cells, telencephalic neurons, and mesenchymal stem cells (MSC‐iPSC) as positive controls. In the wild‐type context, lamin A was not expressed in neurons. The transfection of pre‐miR‐9 in MSC‐iPSC generated a 66% decrease in lamin A protein, whereas lamin C increased by 50%. In HGPS MSC‐iPSC, lamin A and progerin decreased by 35% and 38%, respectively, whereas lamin C increased by 55% after pre‐miR‐9 transfection. Moreover, the abnormal blebs on nuclei decreased by 48% after transfection of pre‐miR‐9 in HGPS MSC‐iPSC, suggesting a protective effect of miR‐9 in neurons of patients with HGPS. They also confirmed the direct targeting of miR‐9 on lamin A 3′UTR using a luciferase reporter assay. Based on this interesting discovery, Fong and colleagues (Jung et al., 2012) hypothesized that an induced ectopic expression of progerin in neurons would lead to neuropathy *in vivo*. They generated the *Lmna* ^*HG‐C*^ mouse model*,* corresponding to the *Lmna* ^HG/+^ mouse, for which the 3′UTR binding site of miR‐9 in prelamin A transcript was removed. Interestingly, these mice, which produced progerin in the brain as expected, did not develop pathology in the central nervous system. Surprisingly, they developed achalasia, a gastrointestinal pathology characterized by a dilated esophagus and reduced amounts of muscle within the *muscularis externa*. The authors suspected that neurons of the central nervous system were less susceptible to the toxicity of progerin than the gastrointestinal tract (Yang et al., 2015). All these studies demonstrate that miR‐9 is a brain‐specific miRNA physiologically inhibiting lamin A expression in neurons, which could explain the absence of neuronal disorders in patients with HGPS by preventing progerin accumulation and its associated toxic effects.

Arancio et al. used a bioinformatics approach to perform a competing endogenous RNA analysis (ceRNAs; Arancio, 2012; Arancio, Giordano & Pizzolanti, 2013). This method predicts RNAs that could be concomitantly regulated because they share common miRNA target sequences in their 3′UTR; importantly, wild‐type prelamin A and progerin share the same 3′UTR. Using miRWalk database, they identified eleven microRNAs in *Homo sapiens* predicted to target the 3′UTR of the prelamin A transcript: miR‐9, miR‐34a, miR‐214, miR‐298, miR‐342‐5p, miR‐449a, miR‐532‐3p, miR‐539, miR‐608, miR‐637, and miR‐671‐5p. As expected, miR‐9 belonged to this list. Second, they listed all the predicted RNAs potentially targeted by these 11 miRNAs. They considered RNAs as ceRNAs if they shared at least three target miRNAs with prelamin A mRNA. Seventeen gene transcripts were considered as potential ceRNAs as they are predicted to be co‐regulated with prelamin A mRNA. Surprisingly, four belong to the miRNA processing machinery: *DICER1* encoding Dicer, *RNEASEN* encoding Drosha, and *EIF2C1* and *EIF2C2* encoding Argonaute proteins. However, the relationship between these proteins and lamin A needs to be clarified. Interestingly, the authors also described a putative co‐regulation of mRNAs involved in the cell cycle (*TP53*,* CDKN1A*,* CDC25A,* and *CDK6*) or inflammation and angiogenesis (*NFKB1, IL1B,* and *VEGFA*). Finally, these predictions of ceRNAs must be experimentally confirmed, and their potential roles in HGPS, and more largely in laminopathies, need to be clarified.

Restrictive dermopathy (OMIM: #275210) is another laminopathy associated with accelerated aging features. It could be considered as the most severe laminopathy identified thus far, as it has a neonatal fatal issue, manifesting initially with severe intrauterine growth retardation among other symptoms. This pathology is caused either by dominant *de novo LMNA* mutations or more frequently by recessive null (homozygous or compound heterozygous) *ZMPSTE24* mutations. Depending on the implicated mutation, a farnesylated truncated or wild‐type full prelamin A accumulates in nuclei (Navarro et al., 2004). The *Zmpste24*‐deficient mouse model *(Zmpste24* ^*−/−*^ *)* presents severe growth retardation and premature death associated with cardiomyopathy, muscular dystrophy, and lipodystrophy (Pendás et al., 2002). In these mice, farnesylated prelamin A accumulates, as expected, but the phenotype corresponds to human HGPS rather than restrictive dermopathy. For this reason, it has long been considered as a good mouse model to study HGPS and associated syndromes linked to prenylated prelamin A accumulation. Even though no miRNA has been formally described in human restrictive dermopathy, several studies have examined miRNAs in the *Zmpste24* ^*−*/*−*^ mouse model or derived cells *in vitro*. López‐Otín and colleagues performed a miRNA expression study on this mouse model and revealed the role of the miR‐29 family (miR‐29a, miR‐29b, miR‐29c) in the DNA damage response (Ugalde, Ramsay et al., 2011). These miRNAs were upregulated in the liver and muscle of *Zmpste24* ^*−*/*−*^ mice and in tissue samples of aged wild‐type mice. They demonstrated that miR‐29 expression progressively increased *in vitro* during passages in *Zmpste24* ^*−*/*−*^ mouse fibroblasts and in wild‐type mouse fibroblasts, whereas no increase was observed in *p53* ^*−/−*^ mouse fibroblasts. They observed that miR‐29 overexpression led to reduced cell proliferation and induction of cell senescence. Using a luciferase reporter assay, they identified the mRNA encoding Ppmd1/Wip1 phosphatase, a key regulator of the DNA damage response that dephosphorylates p53, as a direct target of miR‐29. They proposed a model in which increased miR‐29 represses Ppm1d, converging in p53 signaling activation. The same team identified miR‐1 as a second microRNA upregulated in the *Zmpste24* ^*−*/*−*^ mouse liver and in cultured fibroblasts of patients with HGPS. They showed that miR‐1 repressed insulin‐like growth factor‐1 (IGF‐1) and contributed to somatotroph axis repression by reducing IGF‐1 synthesis (Mariño et al., 2010). Finally, López‐Otín and colleagues introduced an elegant definition of “GeromiRs” as miRNAs involved in pathways linked to aging (Ugalde, Español & López‐Otín, 2011). Xiong et al. (2015) generated small RNA libraries from wild‐type and *Zmpste24* ^*−*/*−*^ mouse embryonic fibroblasts (MEF). They identified 10 differentially expressed miRNAs, among which three were upregulated and eight downregulated. They focused their attention on two downregulated miRNAs, miR‐342‐5p and miR‐365. Xiong and Zhang teams respectively found that these two miRNAs promoted cell proliferation and decreased cell senescence in *Zmpste24* ^*−*/*−*^ MEFs transfected with mimics (Xiong et al., 2015; Zhang et al., 2017). Moreover, miR‐342‐5p overexpression increased G2 + M cell cycle phases. They identified Rasd1, a member of the Ras small GTPase superfamily, as the direct target of miR‐365. Thus, the downregulation of miR‐365 in *Zmpste24* ^*−*/*−*^ MEFs could lead to the overexpression of Rasd1, which has been described as suppressing proliferation and promoting apoptosis (Xiong et al., 2015). Zhang et al. identified GAS2 as a direct target of miR‐342‐5p. Interestingly, GAS2 is a p53‐stabilizing protein involved in p53‐induced growth inhibition, which is a prerequisite for cellular senescence. The authors concluded that miR‐342‐5p promotes cell proliferation and the cell cycle by inhibiting GAS2. Thus, these studies using the *Zmpste24* ^*−*/*−*^ mouse model suggest that the four miRNAs (miR‐1, the miR‐29 family, miR‐342‐5p, and miR‐365) could play a significant role in the pathophysiology of aging diseases linked to an accumulation of prenylated prelamin A, which remains to be confirmed in human cell models.

Atypical Werner syndrome (AWS) is a laminopathy associated with accelerated aging, which differs from the typical Werner syndrome by the causal mutated gene and part of the clinical phenotype. Typical Werner syndrome (OMIM: #277700) is an autosomal‐recessive progeroid disorder manifesting in adulthood that is caused by mutations in *WRN* encoding REQL2, a member of the RECQ family of DNA helicases. AWS is an autosomal‐dominant disease caused by a heterozygous mutation in *LMNA* (Chen et al., 2003). The initial symptoms of premature aging in AWS begin classically 6 years earlier than in the typical form (e.g., loss of hair, bilateral cataract, and scleroderma‐like skin changes). Although AWS shares many common symptoms with the typical form, other symptoms are specific to each form. To date, no miRNA has been formally described as deregulated in AWS. However, in the context of the typical Werner syndrome, Dallaire et al. (2012) reported the downregulation of miR‐124 in the liver of a 3‐month‐old mouse carrying a deletion in the helicase domain of the murine *WRN* homologue (*Wrn* ^Dhel/Dhel^) and in the *C. elegans wrn‐1* mutant (Dallaire et al., 2012). They also demonstrated that the loss of miR‐124 generated by a deletion of the *miR‐124* gene in *C. elegans* increased reactive oxygen species (ROS), reduced ATP production, accelerated the accumulation of lipofuscin (an aging marker), and ultimately reduced the lifespan. As it was also reduced in the liver of aged wild‐type mice compared to young mice, they proposed miR‐124 downregulation as a common signature in the liver of aging mice. Conversely, Tang et al. (2016) reported differentially expressed miRNAs in fibroblasts of patients with Werner syndrome compared to controls. Using ingenuity pathway analysis, they identified 18 miRNAs that were linked to 218 target genes that may contribute to Werner syndrome pathophysiology (Tang et al., 2016). It could be interesting to study the role of the best candidates potentially linked to the aging features and evaluate these miRNAs in the context of atypical Werner syndrome.

Finally, several studies have focused on the role of miRNAs during physiological aging, which enter in the field of “geromiRs” as already discussed above. In this context, miRNAs can regulate prelamin A/lamin A expression to influence physiological aging (Yu et al., 2013). Yu et al. studied the role of miR‐141‐3p, which increases during replicative senescence in human mesenchymal stem cells (hMSCs). They demonstrated *in vitro* that this increase during passages led to a progressive decrease in its direct target FACE1/ZMPSTE24 mRNA, which in turn led to prelamin A accumulation in these cells. Moreover, miR‐141‐3p overexpression induced senescence in wild‐type hMSCs derived from bone marrow and adipose tissue. They also confirmed *in vivo* the decrease in ZMPSTE24 in the liver of wild‐type mice after intraperitoneal injection of miR‐141‐3p lentivirus. They proposed that the increase in miR‐141‐3p could be due to epigenetic modifications, inducing a decrease in histone deacetylase HDAC2 and HDAC1 expression. Interestingly, miR‐141‐3p has been implicated in the pathophysiology of Dunnigan‐type familial partial lipodystrophy, a laminopathy described as being associated with a prelamin A accumulation (see below).

### 3.2 miRNAs in lipodystrophic syndromes

Mutations in *LMNA* also cause adipose tissue pathologies. The autosomal‐dominant Dunnigan‐type familial partial lipodystrophy (FPLD2, OMIM: #151660) is characterized by an abnormal subcutaneous adipose tissue distribution, with a loss of subcutaneous adipose tissue from the trunk, buttocks, and limbs, and fat accumulation in the neck and face. Metabolic complications, such as diabetes mellitus, insulin resistance, hypertriglyceridemia, premature atherosclerosis, and cardiovascular events, are usually observed (Araújo‐Vilar et al., 2009; Shackleton et al., 2000). Oldenburg et al. (2017) identified the promyogenic and antiadipogenic miRNA, miR‐335, as upregulated in fibroblasts from FPLD2 patients and primary adipose stem cells (ASCs) carrying the *LMNA* hotspot mutation in this disease, p.R482W. They demonstrated that miR‐335 upregulation was caused by epigenetic modifications. Indeed, under basal conditions, they observed a punctual lamin A/C binding to the *MIR335* locus in ASCs from controls. After adipogenic induction, the increase in H3K27 trimethylation coincided with lamin A/C binding to the *MIR335* locus, inducing a repression of miR‐335, which allowed adipocyte differentiation. Conversely, in FPLD2 ASCs, lamin A/C mutation prevented binding to the *MIR335* locus under basal conditions and after adipogenic induction. In FPLD2 cells, the acetylation of enhancers favored an increase in miR‐335 transcription, and the resulting overexpression of miR‐335 prevented the differentiation of ASCs into mature adipocytes. Therefore, this epigenetic modification could at least in part explain the lipodystrophic phenotype in patients.

As Yu et al. (2013) showed that miR‐141‐3p decreases ZMPSTE24 expression, resulting in an accumulation of prelamin A during physiological aging (see paragraph above; Yu et al., 2013), and Afonso et al. (2016) studied this phenomenon using lipodystrophic models. They previously demonstrated that the p.R842W *LMNA* mutation induced endothelial dysfunction *in vitro* and early atherosclerosis in FPLD2 patients (Bidault et al., 2013). They confirmed the decrease in ZMPSTE24 in parallel to the increase in prelamin A in two models: (i) fibroblasts of lipodystrophic/lipoatrophic patients carrying a mutation in *LMNA* (p.R482W, p.D47Y, or p.R133L) and (ii) in vascular smooth muscle cells (VSMCs) overexpressing these *LMNA* mutations. The p.R842W mutation is frequently identified in FPLD2 patients. The p.R133L mutation has been identified by Caux et al. (2003) in a “generalized lipoatrophy, associated with insulin‐resistant diabetes, disseminated leukomelanodermic papules, liver steatosis, and cardiomyopathy” (LIRLLC). The p.D47Y mutation has been identified in a progeroid syndrome associated with lipodystrophy, dyslipidemia, insulin resistance, and liver steatosis. For these three mutations, the authors found increased ROS production, NF‐ĸB activation, increased mRNA of several pro‐inflammatory cytokines, and decreased anti‐inflammatory cytokine IL‐13 using VSMC models expressing each mutant. Interestingly, premature senescence has also been demonstrated using a β‐galactosidase activity test and p16^INK4^. miR‐141‐3p was increased by more than 1.5‐fold in VSMCs with p.R482W and p.D47Y *LMNA* mutations, whereas no difference was observed with p.R133L compared to the control. However, even if the ZMPSTE24 protein expression decreased, the mRNA level did not. As Yu et al. (2013) confirmed that ZMPSTE24 mRNA was a direct target of miR‐141‐3p using a luciferase reporter assay, the results obtained by Afonso et al. (2016) demonstrated that miR‐141‐3p prevents the translation of ZMPSTE24 mRNA rather than its degradation. Combined, these results indicate that miR‐141‐3p could be a key factor implicated in FPLD2, explaining at least some of the clinical features of aging in this syndrome, particularly vascular premature senescence and its clinical expression associated with complications.

miRNAs, such as miR‐140, can also regulate physiological adipogenesis. Primary adipocyte‐derived stem cells from miR‐140 knock‐out mice lost their adipogenic ability. Furthermore, miR‐140 positively regulates the expression of NEAT1, one of the most overexpressed long‐noncoding RNAs during adipogenesis, by enhancing its stability in the nucleus. The re‐expression of NEAT1 in adipocyte‐derived stem cells rescued the adipogenic phenotype (Gernapudi et al., 2016).

### 3.3 miRNAs in striated muscle diseases

Several laminopathies affect striated muscles, which include skeletal muscle and/or cardiac muscle. Among them, Emery–Dreifuss muscular dystrophy (EDMD) and limb‐girdle muscular dystrophy 1B (LGMD1B) are two dystrophic muscle pathologies presenting with muscle weakness and wasting and clinically differ in the localization of the affected muscles. They are both associated with dilated cardiomyopathy (Bonne et al., 1999; Muchir et al., 2000; Raffaele Di Barletta et al., 2000). Several types of EDMD laminopathies have been described: an autosomal‐dominant form linked to *LMNA* (EDMD2, OMIM #181350), an autosomal‐recessive form linked to *LMNA* (EDMD3, OMIM #616516), and an X‐linked form due to the *EMD* mutation (EDMD1, OMIM #616516) encoding the nuclear envelope protein emerin, which is a direct partner of lamins. Additionally, three other forms (EDMD 4, 5, and 7) implicate other nuclear envelope proteins (SYNE1, SYNE2, and TMEM43). Another laminopathy called congenital muscular dystrophy (autosomal dominant, OMIM #613205) causes muscle weakness from the first year of life, which could be associated with a “dropped head” syndrome phenotype (Quijano‐Roy et al., 2008). Two other laminopathies have a cardiac phenotype. The autosomal‐dominant dilated cardiomyopathy 1A linked to *LMNA* (OMIM #115200) is characterized by an isolated cardiac impairment, while the dilated cardiomyopathy is accompanied by a brachydactyly in hands and feet in the autosomal‐dominant Slovenian type “Heart‐hand” syndrome (OMIM #610140; Fatkin et al., 1999; Renou et al., 2008). Several microRNAs have been described as muscle‐specific and to regulate muscle function, such as proliferation, differentiation, or contractility (Agarwal et al., 2008; van Rooij et al., 2008). In skeletal and cardiac muscles, miR‐1 and miR‐133 regulate cell proliferation and differentiation among other effects. miR‐206 promotes skeletal muscle differentiation, and miR‐208 plays a role in the regulation of the myosin heavy chain.

Sylvius et al. (2011) performed miRNA expression profiling of muscle biopsies of five patients suffering from skeletal muscle dystrophy and/or cardiac disorders (dilated cardiomyopathy, LGMD1B and EDMD) and carrying an *LMNA* mutation (p.R60G, p.R294Q, p.R321X, or p.R377C) as compared to four healthy controls. Among the 667 miRNAs analyzed, 205 were considered as expressed. The unsupervised hierarchical clustering of these miRNAs allowed the separation between normal and *LMNA* mutated biopsies and identified a miRNA signature. Sixteen miRNAs were upregulated in patients, including eight already described in other studies as upregulated in other muscle or cardiac disorders (Eisenberg et al., 2007; e.g., Duchenne muscular dystrophy and limb‐girdle muscular dystrophy types 2A and 2B). The authors focused their attention on miR‐100, miR‐192, and miR‐335, which were also found to be highly expressed in fetal skeletal muscle. In skeletal muscle cells, proliferation and differentiation are mutually exclusive. Using transfection of mimics in C2C12 mouse myoblasts, they demonstrated that the upregulation of miR‐100 could be implicated during myoblast differentiation, whereas miR‐192 and miR‐335 seemed to repress differentiation and induced myoblast proliferation. Therefore, these miRNAs should be considered as integral components of the regulation of muscle development, and their deregulation could thus be implicated in skeletal muscle dystrophy and/or cardiac disorders.

Vignier et al. (2013) searched for miRNA deregulation in the serum of five mouse models of striated muscle pathologies: EDMD, LGMD types 2C and 2D, Duchenne muscular dystrophy, and hypertrophic cardiomyopathy. Among them, only EDMD was classified as laminopathy (mutations implicated in LGMD2C and 2D concerned gamma‐sarcoglycan gene and alpha‐sarcoglycan gene, respectively). The EDMD mouse model was a knock‐in carrying the p.H222P *LMNA* mutation. The authors performed a first miRNome by screening 517 miRNAs and a second by validating 87 of the miRNAs selected in this first miRNome, including miRNAs known to be expressed in normal and pathological muscle. In the EDMD mouse model, two miRNAs, miR‐200a and miR‐146b, were upregulated, and six were downregulated (miR‐1, miR‐130a, miR‐133a, miR‐133b, miR‐151‐3p, and miR‐339‐3p). Concerning miR‐200a, deregulation of this miRNA was not specific to the EDMD mouse model, as it was also upregulated in the hypertrophic cardiomyopathy model, whereas it was downregulated in the three other models of muscular dystrophy. Among the six downregulated miRNAs in the EDMD mouse model, miR‐1, miR‐133a, and miR‐133b (three muscle‐specific miRNAs; Agarwal et al., 2008; van Rooij et al., 2008) were upregulated in the three muscular dystrophy models linked to the dystrophin‐associated protein complex and characterized by massive muscle fiber destruction (Duchenne muscular dystrophy, LGMD2C and 2D). Finally, only miR‐130a and miR‐339‐3p were specifically downregulated in the EDMD mouse model and could therefore be specific biomarkers for this pathology. However, a limitation of this study is that miRNomes were performed using a pool of sera from several mice. Thus, the downregulation of miR‐130a and miR‐339‐3p needs to be confirmed independently in several mice. Moreover, the deregulated miRNAs identified in the sera of EDMD mouse models were different from those identified as deregulated in muscle biopsies from patients with EDMD (Sylvius et al., 2011) and could be linked to other processes not directly related to the muscle pathology.

Koch and Holaska (2012) studied miRNA expression in X‐linked Emery–Dreifuss muscular dystrophy, which is caused by mutations in emerin (*EMD*). They performed miRNA expression profiling on emerin‐null mouse myogenic progenitor cells compared to wild‐type controls. Interestingly, they found an upregulation of miR‐100, which was already described by Sylvius et al. (2011)*,* in muscle biopsies of patients suffering from skeletal muscle dystrophy and/or cardiac disorders linked to *LMNA* mutations. Contrarily, miR‐192, which was upregulated in Sylvius et al. (2011), was found to be downregulated in this study. Using mRNA profiling, this study on myogenic progenitors derived from emerin‐null mice also identified disruptions in the Notch, Wnt, TGF‐β, and IGF pathways, but the authors did not link these defects to miRNA deregulation. Thus, it would be interesting to integrate the data (miRNome and transcriptome) in a systems biology approach in the future.

Finally, DICER deletion in the heart leads to dilated cardiomyopathy followed by death in the first days of life (Chen et al., 2008). These data suggest that miRNAs are fundamental for normal cardiac development. miRNA expression has been studied in several congenital heart diseases (e.g., hypoplastic heart syndrome, tetralogy of Fallot, ventricular septal defects, and Holt–Oram syndrome; Hoelscher et al., 2017). However, no study has focused on dilated cardiomyopathy type 1A and “Heart‐Hand” syndrome linked to lamins.

### 3.4 miRNAs in peripheral neuropathy

Charcot–Marie–Tooth (CMT) disease is an inherited neuromuscular disorder presenting clinical and genetic heterogeneity. The disease is characterized by sensory and motor neuropathies (e.g., muscle weakness, foot deformities, and electrophysiological changes). The autosomal‐recessive form of axonal CMT linked to *LMNA* is classified as type 2B1 (OMIM #605588; De Sandre‐Giovannoli et al., 2002). No miRNA profiling has been performed in this subtype, which is classified as laminopathy.

Another form of CMT type 2 (CMT2F, OMIM #606595) is due to mutations in *HSPB1*, encoding HspB1, also called Hsp27, which is a small heat shock protein (Houlden et al., 2008). In this form, overexpression of miR‐20a and miR‐128 has been associated with decreased PDZ‐RhoGEF regulating RhoA activity, which could lead to the deregulation of neurite growth (Sun, Zhou, Fink & Mata, 2013).

### 3.5 miRNAs in laminopathy affecting LMNB1

Lamins B1 and B2 have been described to play an important role during brain development, especially in neurons (Young, Jung, Coffinier & Fong, 2012). The autosomal‐dominant leukodystrophy (ADLD, OMIM **#**169500) is the only laminopathy currently linked to the gene *LMNB1,* mainly caused by its duplication (Padiath et al., 2006). Clinically, this disease is a progressive degenerative neurological disorder with cerebellar, autonomic, and pyramidal abnormalities. White matter degeneration with severe myelin loss is observed in the brain with the preservation of oligodendroglia. Lamin B1 plays a key role in neuronal migration, as has been shown in different mouse models. It has been evidenced that a lack of lamin B1 expression causes defects in the division of neural progenitor cells, accelerates cell cycle exit, and enhances apoptosis in the cerebral cortex (Lin, Heng, Ptáček & Fu, 2014). Conversely, the overexpression of lamin B1 leads to demyelination and perturbation of the inner nuclear membrane proteins, nuclear pore transport, and chromatin organization in neural cell lines. Therefore, the overexpression of lamin B1 in the mouse brain reproduces the symptoms observed in patients with ADLD.

Lin et al. (2013) provided new mechanisms of lamin B1 regulation, implicating miR‐23a (Lin & Fu, 2009; Lin et al., 2013). They first demonstrated that lamin B1 expression was developmentally regulated as the protein decreased in the mouse brain from birth to 300 days of age, while miR‐23a levels progressively increased in parallel. They demonstrated that miR‐23a directly targets the 3′UTR of lamin B1 and abrogates the adverse effects of lamin B1 on oligodendrocytes. Using a transgenic mouse model overexpressing murine miR‐23a (mmu‐miR‐23a) specifically in oligodendrocytes, they confirmed that miR‐23a enhances myelin synthesis. Furthermore, using an RNA‐Seq approach on these mice, they demonstrated that in addition to lamin B1, miR‐23a also targets *PTEN* and a long‐noncoding RNA (lncRNA) called “2700046G09Rik.” They proposed a model whereby miR‐23a is a central player in the regulation of myelination. Lamin B1 overexpression decreased myelin gene transcription, generated a myelin mislocalization and decreased its production. miR‐23a increases myelination by repressing lamin B1 expression. Furthermore, miR‐23a enhances 2700046G09Rik and represses PTEN in oligodendroglia, leading to AKT activation, which promotes myelination (Lin et al., 2014). This interesting work conducted by Lin and colleagues raised several issues. As miR‐23a seems to play a key role in myelination regulation, it would be interesting to compare the expression of this miRNA in patients with ADLD and healthy subjects. Moreover, this study provides a new potential therapeutic approach for ADLD.

Dreesen et al. (2013) confirmed *in vitro* the role of miR‐23a on lamin B1 expression using fibroblasts. They demonstrated that lamin B1 transcripts decreased by ≈20‐fold in senescent fibroblasts compared to proliferating fibroblasts, whereas lamin A/C transcripts remained stable. Concomitantly, they found that miR‐23a was increased by ≈2.5‐fold in the same senescent cells. Finally, the overexpression of miR‐23a in fibroblasts did not significantly reduce lamin B1 mRNA levels but decreased the protein expression by ≈30%, suggesting that miR‐23a acts by blocking translation. On the other hand, they developed an interesting theory concerning central nervous system injury in patients with ADLD. They demonstrated that cells presenting decreased lamin A and/or C expression (detected using an antibody against a common epitope) are more susceptible to variations of lamin B1 expression, as an increase in lamin B1 in cells with reduced lamins A and/or C expression led to a proliferative defect, telomeric DNA damage, and increased senescence. Therefore, the brain is more susceptible to B‐type fluctuations due to the low expression of lamin A in neurons. This hypothesis could explain why, in ADLD, neurons overexpressing lamin B1 but poorly expressing lamin A due to miR‐9 expression (Jung et al., 2012) are more affected than other cell types.

mir‐351 has also been described to modulate lamin B1 expression using an F28‐7 cell model not related to laminopathy (a clone of mouse mammary carcinoma FM3A). Sato et al. (2016) investigated the molecular mechanisms regulating apoptosis and necrosis. They demonstrated that miR‐351 decreased lamin B1 after mimic transfection. However, as lamin B1 is not a validated target of miR‐351, an indirect downregulation could not be excluded.

To understand the involvement of the nuclear envelope and *lamina* and more specifically B‐type lamins in miRNA regulation, Malhas, Saunders and Vaux (2010) performed microRNA expression profiling in MEFs lacking the C‐terminus of lamin B1 (*Lmnb1* ^Δ/Δ^ cells). They found four downregulated and 18 upregulated miRNAs (including miR‐351) compared to wild‐type MEFs. miR‐31 was the most upregulated miRNA. According to previously published data, they concluded that lamin B1 could sequester the Oct‐1 transcription factor in wild‐type MEFs, leading to a low expression of miR‐31. In contrast, the truncation of lamin B1 in *Lmnb1* ^Δ/Δ^ MEFs could lead to an increase in Oct‐1, resulting in an increase in miR‐31 expression. They identified *Cdkn2a* as a direct target of miR‐31. This gene encodes two proteins, p16^Ink4a^ and p19^arf^, which are involved in the cell cycle negative regulation. The decrease in these proteins in *Lmnb1* ^Δ/Δ^ MEFs led to rapid progress from G1 to S‐phase. Therefore, the truncation at the C‐terminus of lamin B1 leads to positive regulation of the cell cycle via the downregulation of p16^Ink4^ and p19^Arf^ secondary to miR‐31 overexpression. Taken together, these studies demonstrate that miRNAs can modulate lamin B1 expression; inversely, the modification of this protein can also impact the expression of miRNAs. These mechanisms could play a significant role in physiological pathways but also in the pathological context of laminopathies.

## 4 miRNAs AND FUTURE PERSPECTIVES IN LAMINOPATHIES

The recent enhanced knowledge of the role of miRNAs in laminopathies has led to a better understanding of their pathophysiology and more largely of the physiological roles of lamins. The best examples are the inhibition of lamin A expression by miR‐9 and lamin B1 by miR‐23a. Better understanding the roles of miRNAs in laminopathies paves the way to new therapeutic approaches in these rare but also sometimes dramatic diseases (van Rooij & Kauppinen, 2014). The recent example of miR‐122, which is the first miRNA targeting approach evaluated with success in a clinical trial (NCT01200420), indicated the possible use of this approach. This liver‐specific miRNA recognizes the 5′UTR of the Hepatitis C virus (HCV) genome, which stabilizes the HCV RNA, favoring virus replication. The antagomiR miravirsen^®^ is a locked nucleic acid‐modified phosphorothioate oligonucleotide targeting miR‐122, which has been shown to induce a decrease in HCV RNA levels. To our knowledge, the clinical trial is still in phase 2 (Janssen et al., 2013; van der Ree et al., 2016). Several other therapeutics targeting miRNAs are currently being evaluated, many in the field of cancer, opening new, encouraging therapeutic perspectives, even if limiting factors for the use of these approaches exist.

In HGPS, should we imagine treating patients with miR‐9 to repress progerin expression in affected tissues and organs (Harhouri et al., in press)? Several limitations of the use of this potential therapeutic approach shall be taken into consideration. The most important limitation is the low specificity of these small RNA molecules on targets, as one microRNA could inhibit several mRNAs, leading to a high theoretical risk of off‐target effects. For example, miR‐9 is predicted to target 1377, 545, and 683 mRNAs using TargetScan7.1 (Agarwal, Bell, Nam & Bartel, 2015), miRDB (Wong & Wang, 2015) and PicTar (Krek et al., 2005), respectively; to date, up to 360 validated targets are listed in miRTarBase (Chou et al., 2016). Therefore, it will be imperative to evaluate the off‐target effects of this miRNA approach or others, *in silico*,* in vitro* in cell models and *in vivo* in animals before progressing to humans.

## 5 CONCLUSION

MicroRNAs have been shown to play crucial roles in physiology and pathologies related to lamins. This class of small noncoding RNAs influences many pathways dysregulated in laminopathies. To date, few studies have focused on miRNAs in these diseases, although the role of these small molecules could be central to their pathophysiology. Future works on miRNAs in laminopathies could provide new insights into these pathologies and perhaps lead to new therapeutic approaches with the ultimate goal of treating patients.

## ACKNOWLEDGMENTS

This work was supported by Institut National de la Santé et de la Recherche Médicale (INSERM), Aix‐Marseille University, A*Midex Foundation (VinTAGE Program) and the Association Française contre les Myopathies (AFM grant TRIM‐RD 2016‐2020 to NL). This study is part of the FHU A*MIDEX project MARCHE n.ANR‐11‐IDEX‐001‐02 funded by the “Investissement d'avenir” French governmental program, managed by the French National Research Agency (ANR).

## CONFLICT OF INTEREST

None declared.

## AUTHOR CONTRIBUTIONS

All authors wrote at least a significant part of the review, approved the version to be published, and agreed to be accountable for all aspects of the work.
